# Functional associations among G protein-coupled neurotransmitter receptors in the human brain

**DOI:** 10.1186/1471-2202-15-16

**Published:** 2014-01-17

**Authors:** Skirmantas Janušonis

**Affiliations:** 1 Department of Psychological and Brain Sciences, University of California, Santa Barbara, CA, USA

**Keywords:** Human brain, G protein-coupled receptor, mRNA, Network, Communities, Receptor sets

## Abstract

**Background:**

The activity of neurons is controlled by groups of neurotransmitter receptors rather than by individual receptors. Experimental studies have investigated some receptor interactions, but currently little information is available about transcriptional associations among receptors at the whole-brain level.

**Results:**

A total of 4950 correlations between 100 G protein-coupled neurotransmitter receptors were examined across 169 brain regions in the human brain using expression data published in the Allen Human Brain Atlas. A large number of highly significant correlations were found, many of which have not been investigated in hypothesis-driven studies. The highest positive and negative correlations of each receptor are reported, which can facilitate the construction of receptor sets likely to be affected by altered transcription of one receptor (such sets always exist, but their members are difficult to predict). A graph analysis isolated two large receptor communities, within each of which receptor mRNA levels were strongly cross-correlated.

**Conclusions:**

The presented systematic analysis shows that the mRNA levels of many G protein-coupled receptors are interdependent. This finding is not unexpected, since the brain is a highly integrated complex system. However, the analysis also revealed two novel properties of global brain structure. First, receptor correlations are described by a simple statistical distribution, which suggests that receptor interactions may be guided by qualitatively similar processes. Second, receptors appear to form two large functional communities, which might be differentially affected in brain disorders.

## Background

A typical neuron receives thousands of synaptic contacts [1], and each postsynaptic site can express a number of neurotransmitter receptors. Since neurons integrate signals from all receptors on their surface, their activity is determined by receptor sets and not by individual receptors.

Functional receptor groups explain why a constitutive null-mutation of a neurotransmitter receptor often produces a mild phenotype, even when the receptor is known to be important in specific brain functions [2-10]. Such minor functional effects can be explained by compensatory mechanisms in the developing brain, which at least partially depend on receptors that detect other neurotransmitters [4,11].

Similarly, the absence of an entire neurotransmitter may not have a major effect on brain development and function. Serotonin (5-hydroxytryptamine, 5-HT) is an extremely abundant neurotransmitter in the brain: by some estimates, the density of serotonergic varicosities in the rat cerebral cortex is around 6·10^6^/mm^3^, with each cortical neuron receiving some 200 varicosities [12]. The density of serotonergic projections may exceed that of the brain capillary system [13,14] and must carry a significant energetic cost. However, genetic mutations and pharmacological manipulations that eliminate virtually all 5-HT in the brain produce only subtle behavioral alterations, with no gross morphological or cellular changes [15-18]. Abnormally low dopamine levels in the brain can be nearly asymptomatic until around 50-80% of the substantia nigra neurons are lost [19], and ablation of dopaminergic neurons in neonatal rats does not result in any significant motor dysfunctions [20,21]. A lack of norepinephrine due to a genetic mutation in the dopamine-β-hydroxylase gene produces an unremarkable neurological phenotype in humans [22,23]. Mammalian thalamic nuclei can function normally in the virtual absence of GABAergic interneurons, as has been shown in mice and rats [24,25]. Experimental evidence shows that the lack of one neurotransmitter can be compensated for by changes in other neurotransmitters: for example, serotonergic processes have been shown to permanently extend into brain areas previously occupied by dopaminergic terminals [26], and serotonin and/or dopamine may compensate for the lack of norepinephrine [27,28]. It should be noted that at least some adult neurons have the flexibility to switch from one neurotransmitter to another in response to the environment [29].

These findings suggest that biological information in the brain is coded not by individual neurotransmitters or their receptors, but by finely-tuned neurotransmitter-receptor sets. While this hypothesis does not require that the receptors be physically linked, it is supported by the unexpected abundance of heteromeric receptor complexes [30,31]. For example, the serotonin 5-HT_2A_ receptor can form complexes with the metabotropic glutamate mGluR_2_ receptor [32] and the dopamine D_2_ receptor [33], which may play a major role in the action of antipsychotic drugs and hallucinogens. A heterocomplex is more than the sum of its individual receptors, since heteromerization can alter receptor mobility at the neuron surface, downstream signaling, and intracellular trafficking [32,34].

Understanding neurotransmitter receptor sets will require new analytical and theoretical approaches. Functionally complete receptor sets have to be isolated and their dynamic properties investigated in specific brain regions and in individual cells [35]. While these studies pose technical challenges, they are likely to lead to major theoretical simplifications. Some researchers have already used this approach with considerable success [36-38]. As a further step toward this goal, the present study used the Allen Human Brain Atlas [39,40] to examine mRNA expression associations among nearly all known G protein-coupled neurotransmitter receptors in the human brain.

## Results

**Figure 1:**
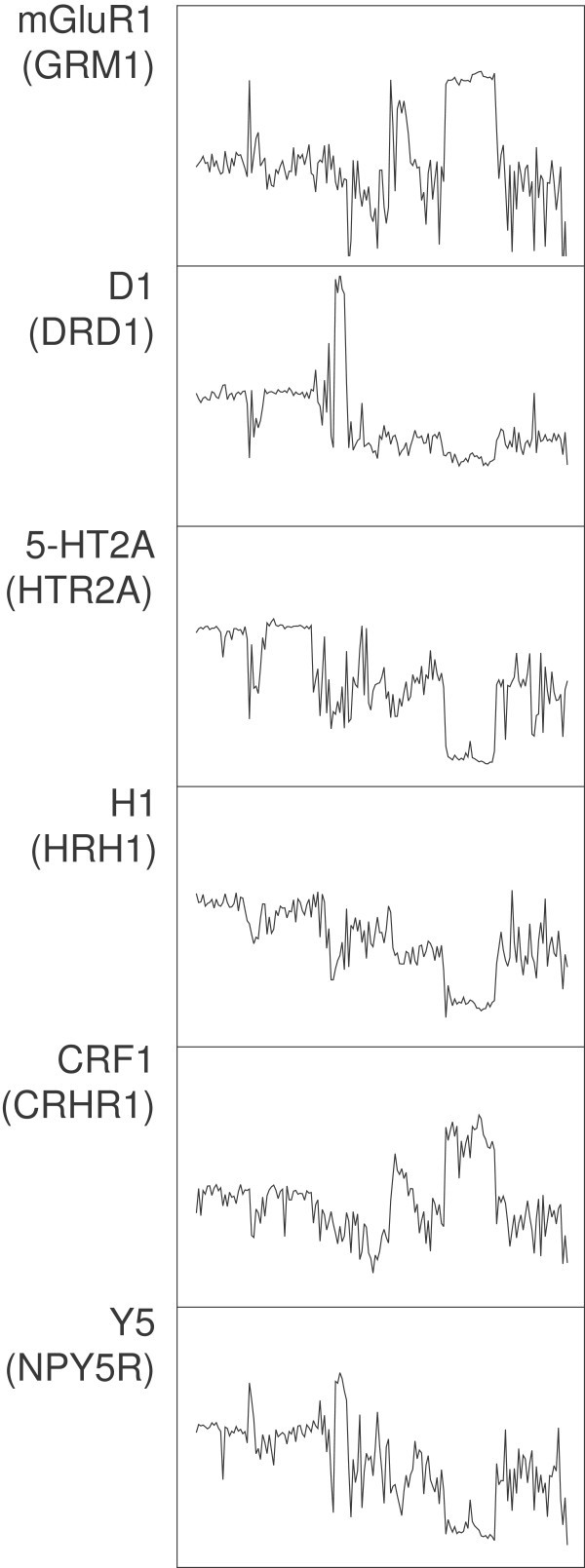
**The mRNA expression profiles of six neurotransmitter receptors.** The horizontal axis represents the 169 brain regions and the vertical axis represents the mRNA amounts (averaged across the probes and subjects). Since the numerical mRNA values are normalized and relative, they are not indicated on the vertical axis (for all six genes, it ranges from −2 to 2). Note high similarity between some of the profiles (e.g., mGluR_1_ and CRF_1_, 5-HT_2A_ and H_1_).

**Figure 2:**
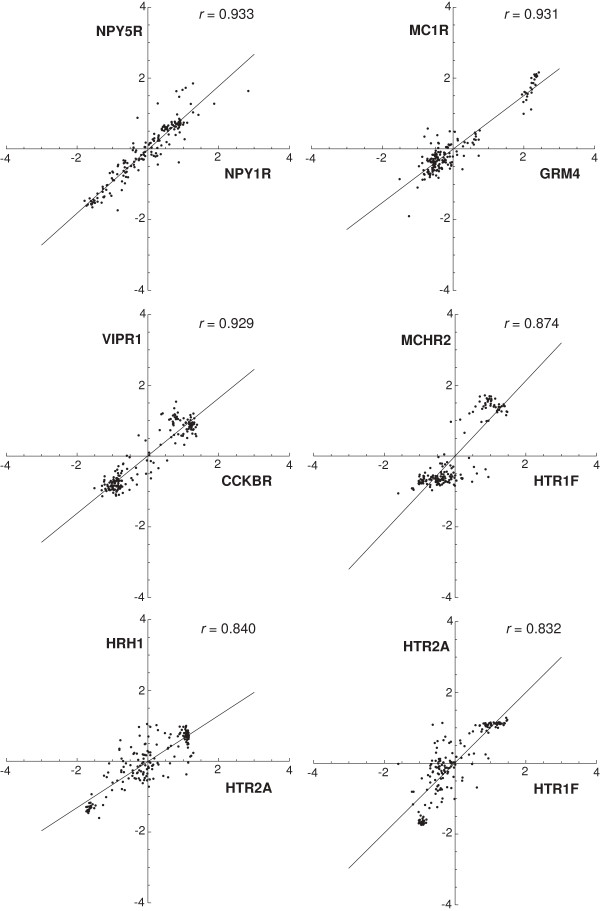
**The scatter plots of the six most strongly correlated receptor pairs (out of the 4950 pairs).** Each point represents the mRNA amounts (averaged across the probes and subjects) of the two receptors in one of the 169 brain regions. Considerable clustering is apparent, which indicates that in most brain regions the mRNA levels of the two receptors are either low or high simultaneously, with few regions in between. All correlations are significant at *p* < 10^-15^.

**Figure 3:**
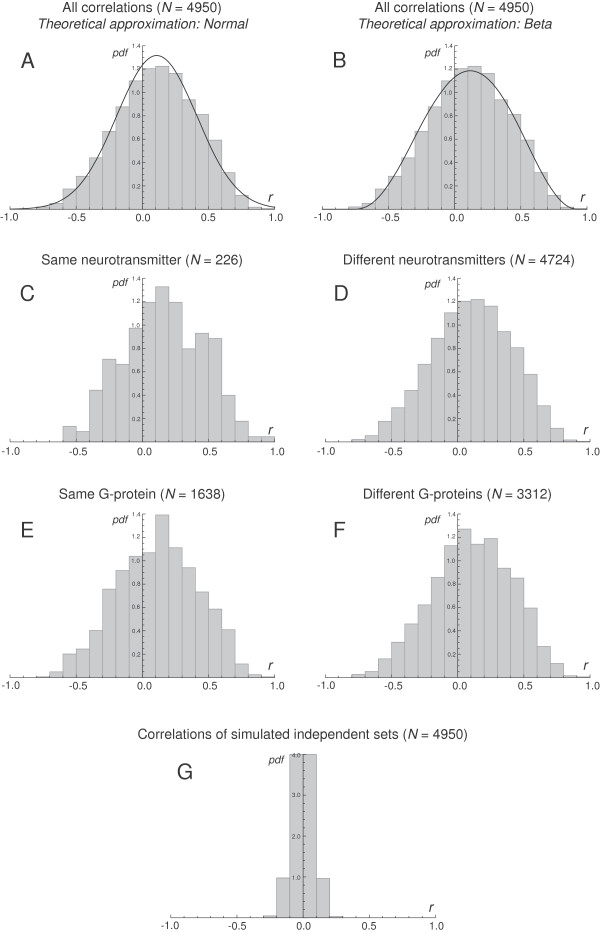
**The statistical distributions of all correlations and their subsets. A**, **B**, The distribution of all correlations and their approximations with the normal distribution **(A)** and the beta distribution **(B)**. Both theoretical distributions have the sample mean and variance. **C**, The distribution of the correlations between receptors that represent the same neurotransmitter (in different pairs, the neurotransmitters may be different). **D***,* The distribution of the correlations between receptors that represent different neurotransmitters. **E***,* The distribution of the correlations between receptors that have the same G protein-coupling (in different pairs, the couplings may be different). **F***,* The distribution of the correlations between receptors that have different G protein-couplings. **G***,* The distribution of the correlations of a simulated sample of 100 sets, each of which contains 169 independent, normally distributed numbers (with mean = 0 and standard deviation = 1). The simulation shows that the distribution of the correlations between G protein-coupled receptors is different from what would be expected from a matching sample of uncorrelated sets.

The analysis used the mRNA expression data of 100 G protein-coupled receptors (Table 1) in 169 regions of six normal human brains presently available in the Allen Brain Atlas database (Figure 1). The mRNA amounts of many receptor pairs were very strongly correlated (Figure 2). The five strongest positive and negative correlations of each receptor are given in Table 2. The distribution of all 4950 correlations had a nearly symmetric shape, with a single mode shifted toward the positive values (Figure 3A, B). This distribution failed normality tests (Figure 3A; the Kolmogorov-Smirnov test: *D* = 0.024, *p* < 0.01; the Shapiro-Wilk test: *W* = 0.995, *p* < 0.01), but was well described by the beta distribution with the same range, mean and variance (the shape parameters *α* = 3.51 and *β* = 3.32) (Figure 3B; *D* = 0.015, *p* = 0.209). The distributions of several functionally meaningful subsets were not significantly different from the complete set (in each pair, both receptors represent the same neurotransmitter (Figure 3C): *D* = 0.076, *p* = 0.169); (in each pair, the receptors represent different neurotransmitters (Figure 3D): *D* = 0.004, *p* = 1); (in each pair, both receptors have the same G protein-coupling (Figure 3E): *D* = 0.015, *p* = 0.950); (in each pair, the receptors have different G protein-couplings (Figure 3F): *D* = 0.007, *p* = 1)).

**Table 1:** The analyzed G protein-coupled receptors

**Number**	**Neurotransmitter**	**Receptor**	**Gene**	**Coupling**	**Median inter-subject correlation**
1	Glutamate	mGluR1	GRM1	Gq	0.91
2	Glutamate	mGluR2	GRM2	Gi	0.37
3	Glutamate	mGluR3	GRM3	Gi	0.82
4	Glutamate	mGluR4	GRM4	Gi	0.94
5	Glutamate	mGluR5	GRM5	Gq	0.92
6	Glutamate	mGluR6	GRM6	Gi	0.12
7	Glutamate	mGluR7	GRM7	Gi	0.89
8	Glutamate	mGluR8	GRM8	Gi	0.45
9	GABA	GABABR1	GABBR1	Gi	0.78
10	GABA	GABABR2	GABBR2	Gi	0.89
11	Dopamine	D1	DRD1	Gs	0.91
12	Dopamine	D2	DRD2	Gi	0.89
13	Dopamine	D3	DRD3	Gi	0.69
14	Dopamine	D4	DRD4	Gi	0.03
15	Dopamine	D5	DRD5	Gs	0.83
16	Adrenergic	α1A	ADRA1A	Gq	0.76
17	Adrenergic	α1B	ADRA1B	Gq	0.87
18	Adrenergic	α1D	ADRA1D	Gq	0.79
19	Adrenergic	α2A	ADRA2A	Gi	0.62
20	Adrenergic	α2B	ADRA2B	Gi	0.31
21	Adrenergic	α2C	ADRA2C	Gi	0.64
22	Adrenergic	β1	ADRB1	Gs	0.59
23	Adrenergic	β2	ADRB2	Gs	0.67
24	Adrenergic	β3	ADRB3	Gs/Gi	0.15
25	Serotonin	5-HT1A	HTR1A	Gi	0.86
26	Serotonin	5-HT1B	HTR1B	Gi	0.38
27	Serotonin	5-HT1D	HTR1D	Gi	0.79
28	Serotonin	5-HT1E	HTR1E	Gi	0.82
29	Serotonin	5-HT1F	HTR1F	Gi	0.90
30	Serotonin	5-HT2A	HTR2A	Gq	0.95
31	Serotonin	5-HT2B	HTR2B	Gq	0.12
32	Serotonin	5-HT2C	HTR2C	Gq	0.92
33	Serotonin	5-HT4	HTR4	Gs	0.89
34	Serotonin	5-HT5A	HTR5A	Gs	0.89
35	Serotonin	5-HT6	HTR6	Gs	0.21
36	Serotonin	5-HT7	HTR7	Gs	0.89
37	Cholinergic	M1	CHRM1	Gq	0.53
38	Cholinergic	M2	CHRM2	Gi	0.91
39	Cholinergic	M3	CHRM3	Gq	0.96
40	Cholinergic	M4	CHRM4	Gi	0.53
41	Cholinergic	M5	CHRM5	Gq	0.63
42	Histamine	H1	HRH1	Gq	0.82
43	Histamine	H2	HRH2	Gs	0.69
44	Histamine	H3	HRH3	Gi	0.66
45	Histamine	H4	HRH4	Gi	0.09
46	Bradykinin	B1	BDKRB1	Gq	0.69
47	Bradykinin	B2	BDKRB2	Gq	0.61
48	Cholecystokinin	CCK1	CCKAR	Gq	0.46
49	Cholecystokinin	CCK2	CCKBR	Gq	0.94
50	CRH	CRF1	CRHR1	Gs	0.65
51	CRH	CRF2	CRHR2	Gs	0.35
52	Galanin	Gal1	GALR1	Gi	0.74
53	Galanin	Gal2	GALR2	Gi/Gq	0.54
54	Galanin	Gal3	GALR3	Gi	0.08
55	MCH	MCH1	MCHR1	Gi	0.70
56	MCH	MCH2	MCHR2	Gq	0.93
57	MSH	MC1	MC1R	Gs	0.73
58	MSH	MC2	MC2R	Gs	0.10
59	MSH	MC3	MC3R	Gs	0.40
60	MSH	MC4	MC4R	Gs	0.49
61	MSH	MC5	MC5R	Gs	0.04
62	NPY	Y1	NPY1R	Gi	0.91
63	NPY	Y2	NPY2R	Gi	0.74
64	NPY	Y4	PPYR1	Gi	0.18
65	NPY	Y5	NPY5R	Gi	0.86
66	NPY	Y6	NPY6R	Gi	0.36
67	Neurotensin	NT1	NTSR1	Gq	0.48
68	Neurotensin	NT2	NTSR2	Gq	0.81
69	Opioid	μ	OPRM1	Gi	0.84
70	Opioid	δ	OPRD1	Gi	0.25
71	Opioid	κ	OPRK1	Gi	0.67
72	Nociceptin	ORL-1	OPRL1	Gi	0.81
73	Orexin	OX1	HCRTR1	Gq	0.82
74	Orexin	OX2	HCRTR1	Gi	0.64
75	Oxytocin	OT	OXTR	Gq	0.75
76	Somatostatin	SST1	SSTR1	Gi	0.81
77	Somatostatin	SST2	SSTR2	Gi	0.91
78	Somatostatin	SST3	SSTR3	Gi	0.28
79	Somatostatin	SST4	SSTR4	Gi	0.07
80	Somatostatin	SST5	SSTR5	Gi	0.16
81	Tachykinin	NK1	TACR1	Gq	0.55
82	Tachykinin	NK2	TACR2	Gq	0.34
83	Tachykinin	NK3	TACR3	Gq	0.81
84	TRH	TRHR	TRHR	Gq	0.79
85	VIP	VPAC1	VIPR1	Gs	0.86
86	VIP	VPAC2	VIPR2	Gs	0.77
87	Vasopressin	V1a	AVPR1A	Gq	0.34
88	Vasopressin	V1b	AVPR1B	Gq	0.04
89	Vasopressin	V2	AVPR2	Gs	0.16
90	Adenosine	A1	ADORA1	Gi	0.66
91	Adenosine	A2A	ADORA2A	Gs	0.87
92	Adenosine	A2B	ADORA2B	Gs	0.57
93	Adenosine	A3	ADORA3	Gi	0.37
94	Purine	P2Y1	P2RY1	Gq	0.72
95	Purine	P2Y2	P2RY2	Gi/Gq	0.37
96	Purine	P2Y4	P2RY4	Gi/Gq	0.00
97	Purine	P2Y6	P2RY6	Gq	0.32
98	Purine	P2Y11	P2RY11	Gq	0.34
99	Cannabinoid	CB1	CNR1	Gi	0.90
100	Cannabinoid	CB2	CNR2	Gi	0.46

The inter-subject correlations were obtained from 6 subjects (15 cross-correlations).

**Table 2:** The five strongest positive and negative correlations of each receptor with other receptors, calculated using 169 brain regions

**1) mGluR1**		**2) mGluR2**		**3) mGluR3**		**4) mGluR4**		**5) mGluR5**		**6) mGluR6**	
50	0.80	72	0.65	90	0.72	57	0.93	72	0.82	88	0.63
4	0.74	18	0.64	43	0.62	50	0.76	39	0.81	4	0.63
57	0.71	43	0.63	23	0.51	1	0.74	65	0.80	57	0.60
46	0.65	97	0.60	22	0.50	46	0.68	7	0.79	50	0.54
98	0.63	23	0.58	93	0.49	47	0.66	43	0.76	47	0.53
68	−0.51	69	−0.33	6	−0.22	62	−0.64	50	−0.32	42	−0.34
52	−0.53	80	−0.34	59	−0.26	39	−0.64	46	−0.39	39	−0.35
75	−0.54	46	−0.35	69	−0.27	76	−0.68	47	−0.49	32	−0.39
40	−0.55	57	−0.55	57	−0.31	30	−0.72	57	−0.57	22	−0.42
32	−0.63	4	−0.56	4	−0.32	42	−0.76	4	−0.58	23	−0.50
**7) mGluR7**		**8) mGluR8**		**9) GABA1**		**10) GABA2**		**11) D1**		**12) D2**	
5	0.79	15	0.57	10	0.63	9	0.63	65	0.74	40	0.71
72	0.76	74	0.52	92	0.57	49	0.58	21	0.74	35	0.69
62	0.65	53	0.47	1	0.49	17	0.58	22	0.71	68	0.67
65	0.65	40	0.47	98	0.49	39	0.56	43	0.70	75	0.66
42	0.64	38	0.46	50	0.47	34	0.56	33	0.69	100	0.66
50	−0.40	50	−0.34	8	−0.25	54	−0.27	4	−0.31	50	−0.49
57	−0.50	4	−0.35	32	−0.25	27	−0.28	47	−0.32	77	−0.50
46	−0.50	98	−0.39	41	−0.28	12	−0.31	69	−0.32	46	−0.60
4	−0.52	1	−0.39	14	−0.31	83	−0.32	57	−0.34	85	−0.67
47	−0.59	57	−0.43	53	−0.31	32	−0.41	54	−0.35	49	−0.70
**13) D3**		**14) D4**		**15) D5**		**16) α1A**		**17) α1B**		**18) α1D**	
91	0.68	51	0.40	72	0.66	84	0.52	56	0.63	72	0.76
27	0.65	70	0.22	75	0.63	63	0.38	39	0.63	36	0.72
33	0.64	98	0.18	68	0.62	7	0.31	30	0.63	5	0.71
21	0.63	97	0.17	52	0.61	94	0.26	42	0.59	2	0.64
12	0.62	46	0.17	82	0.60	79	0.26	55	0.59	39	0.64
34	−0.19	7	−0.29	1	−0.47	39	−0.14	35	−0.29	47	−0.39
10	−0.22	9	−0.31	46	−0.49	74	−0.16	57	−0.30	80	−0.43
49	−0.24	94	−0.31	50	−0.50	90	−0.18	54	−0.30	46	−0.45
46	−0.27	68	−0.34	57	−0.60	20	−0.22	64	−0.30	4	−0.53
50	−0.30	60	−0.35	4	−0.60	44	−0.31	4	−0.35	57	−0.54
**19) α2A**		**20) α2B**		**21) α2C**		**22) β1**		**23) β2**		**24) β3**	
73	0.63	91	0.50	11	0.74	29	0.74	93	0.68	78	0.69
81	0.50	13	0.43	33	0.67	11	0.71	100	0.62	79	0.67
83	0.49	12	0.42	91	0.65	39	0.71	68	0.60	100	0.63
7	0.48	35	0.40	27	0.65	37	0.69	97	0.59	82	0.61
52	0.47	43	0.33	13	0.63	56	0.69	40	0.58	89	0.57
91	−0.27	80	−0.24	57	−0.25	47	−0.44	6	−0.50	77	−0.19
4	−0.27	49	−0.27	47	−0.26	54	−0.44	85	−0.53	98	−0.20
47	−0.29	25	−0.31	1	−0.29	69	−0.45	34	−0.56	56	−0.22
50	−0.30	34	−0.32	46	−0.32	57	−0.50	4	−0.57	85	−0.30
46	−0.33	77	−0.33	50	−0.34	4	−0.53	57	−0.59	49	−0.31
**25) 5-HT1A**		**26) 5-HT1B**		**27) 5-HT1D**		**28) 5-HT1E**		**29) 5-HT1F**		**30) 5-HT2A**	
39	0.66	40	0.67	21	0.65	65	0.62	56	0.87	42	0.84
62	0.64	100	0.61	13	0.65	5	0.58	30	0.83	29	0.83
42	0.64	82	0.61	94	0.63	62	0.56	86	0.80	39	0.80
30	0.64	78	0.61	40	0.60	39	0.55	55	0.78	55	0.80
7	0.62	35	0.60	33	0.59	43	0.53	39	0.78	56	0.76
20	−0.31	1	−0.30	49	−0.30	14	−0.17	66	−0.35	54	−0.40
47	−0.35	98	−0.32	98	−0.32	95	−0.19	69	−0.39	1	−0.43
54	−0.35	49	−0.38	1	−0.32	57	−0.24	57	−0.46	47	−0.54
57	−0.49	85	−0.39	46	−0.44	4	−0.25	54	−0.48	57	−0.71
4	−0.51	46	−0.46	50	−0.46	54	−0.30	4	−0.49	4	−0.72
**31) 5-HT2B**		**32) 5-HT2C**		**33) 5-HT4**		**34) 5-HT5A**		**35) 5-HT6**		**36) 5-HT7**	
61	0.54	75	0.70	65	0.79	85	0.63	12	0.69	18	0.72
35	0.54	68	0.64	5	0.71	49	0.63	40	0.65	72	0.72
45	0.53	94	0.63	11	0.69	77	0.59	100	0.63	38	0.65
58	0.51	12	0.61	21	0.67	1	0.56	26	0.60	7	0.60
88	0.48	40	0.60	62	0.67	10	0.56	27	0.56	68	0.59
62	−0.16	85	−0.61	46	−0.45	40	−0.44	46	−0.33	57	−0.46
17	−0.18	1	−0.63	57	−0.48	78	−0.47	77	−0.35	80	−0.47
49	−0.21	98	−0.64	50	−0.49	95	−0.47	56	−0.39	49	−0.49
85	−0.21	46	−0.70	4	−0.50	100	−0.54	85	−0.50	85	−0.61
56	−0.24	50	−0.76	47	−0.50	23	−0.56	49	−0.54	46	−0.62
**37) M1**		**38) M2**		**39) M3**		**40) M4**		**41) M5**		**42) H1**	
43	0.73	36	0.65	5	0.81	12	0.71	75	0.80	30	0.84
39	0.70	72	0.63	30	0.80	100	0.70	53	0.65	39	0.80
22	0.69	15	0.59	42	0.80	68	0.69	68	0.63	62	0.77
29	0.67	26	0.57	43	0.78	75	0.68	82	0.61	55	0.74
56	0.65	53	0.53	29	0.78	26	0.67	83	0.59	5	0.73
59	−0.36	46	−0.45	69	−0.40	49	−0.53	1	−0.47	50	−0.43
44	−0.37	99	−0.45	54	−0.44	1	−0.55	46	−0.52	1	−0.45
57	−0.45	80	−0.46	47	−0.44	85	−0.58	50	−0.53	47	−0.54
4	−0.45	77	−0.50	57	−0.62	50	−0.58	49	−0.60	57	−0.72
69	−0.55	85	−0.53	4	−0.64	46	−0.64	85	−0.69	4	−0.76
**43) H2**		**44) H3**		**45) H4**		**46) B1**		**47) B2**		**48) CCK1**	
39	0.78	69	0.42	58	0.56	50	0.78	46	0.76	67	0.67
5	0.76	12	0.39	89	0.54	47	0.76	4	0.66	75	0.53
72	0.73	9	0.39	35	0.53	85	0.70	57	0.63	41	0.52
37	0.73	4	0.36	31	0.53	4	0.68	50	0.61	53	0.49
11	0.70	20	0.29	61	0.52	57	0.66	6	0.53	58	0.46
80	−0.30	16	−0.31	86	−0.19	36	−0.62	62	−0.50	46	−0.26
47	−0.35	29	−0.32	98	−0.24	40	−0.64	52	−0.52	50	−0.33
69	−0.42	55	−0.33	56	−0.24	75	−0.69	30	−0.54	85	−0.34
4	−0.50	37	−0.37	85	−0.30	68	−0.69	42	−0.54	49	−0.35
57	−0.50	97	−0.38	49	−0.31	32	−0.70	7	−0.59	98	−0.38
**49) CCK2**		**50) CRF1**		**51) CRF2**		**52) Gal1**		**53) Gal2**		**54) Gal3**	
85	0.93	1	0.80	14	0.40	15	0.61	75	0.75	64	0.62
56	0.78	46	0.78	85	0.39	68	0.61	73	0.67	78	0.56
77	0.71	4	0.76	86	0.34	60	0.59	41	0.65	100	0.56
98	0.67	57	0.74	49	0.31	73	0.57	83	0.64	24	0.55
46	0.63	98	0.71	22	0.30	32	0.57	82	0.63	89	0.55
41	−0.60	40	−0.58	94	−0.29	57	−0.52	50	−0.48	39	−0.44
75	−0.67	94	−0.61	36	−0.29	1	−0.53	46	−0.49	85	−0.44
100	−0.69	68	−0.66	75	−0.33	50	−0.56	49	−0.58	29	−0.48
12	−0.70	75	−0.68	41	−0.34	46	−0.56	98	−0.59	49	−0.54
66	−0.71	32	−0.76	68	−0.41	4	−0.58	85	−0.63	56	−0.63
**55) MCH1**		**56) MCH2**		**57) MC1**		**58) MC2**		**59) MC3**		**60) MC4**	
30	0.80	29	0.87	4	0.93	45	0.56	60	0.34	52	0.59
29	0.78	49	0.78	50	0.74	35	0.54	69	0.29	32	0.56
42	0.74	30	0.76	1	0.71	61	0.53	44	0.27	68	0.56
39	0.72	39	0.75	46	0.66	31	0.51	41	0.26	75	0.55
56	0.71	86	0.74	47	0.63	100	0.47	84	0.24	15	0.47
47	−0.29	12	−0.43	15	−0.60	86	−0.18	2	−0.24	98	−0.38
44	−0.33	100	−0.44	39	−0.62	56	−0.21	23	−0.24	1	−0.40
69	−0.36	64	−0.46	76	−0.65	85	−0.25	90	−0.25	47	−0.44
57	−0.50	66	−0.55	30	−0.71	98	−0.25	3	−0.26	50	−0.45
4	−0.52	54	−0.63	42	−0.72	49	−0.25	37	−0.36	46	−0.54
**61) MC5**		**62) Y1**		**63) Y2**		**64) Y4**		**65) Y5**		**66) Y6**	
88	0.55	65	0.93	7	0.59	54	0.62	62	0.93	100	0.66
31	0.54	42	0.77	42	0.53	100	0.59	5	0.80	75	0.63
58	0.53	5	0.73	62	0.51	96	0.57	33	0.79	12	0.61
45	0.52	39	0.70	65	0.50	89	0.57	11	0.74	82	0.58
6	0.51	33	0.67	30	0.45	24	0.55	39	0.73	36	0.57
11	−0.12	46	−0.39	46	−0.34	85	−0.33	46	−0.38	46	−0.45
90	−0.14	50	−0.43	57	−0.40	30	−0.36	50	−0.44	77	−0.51
98	−0.14	47	−0.50	50	−0.41	49	−0.39	47	−0.49	56	−0.55
91	−0.14	57	−0.57	47	−0.47	86	−0.40	57	−0.54	85	−0.70
22	−0.16	4	−0.64	4	−0.51	56	−0.46	4	−0.59	49	−0.71
**67) NT1**		**68) NT2**		**69) μ**		**70) δ**		**71) κ**		**72) ORL-1**	
48	0.67	75	0.81	4	0.49	43	0.61	72	0.52	5	0.82
75	0.65	94	0.77	57	0.44	22	0.60	62	0.51	7	0.76
68	0.64	40	0.69	44	0.42	37	0.59	65	0.48	18	0.76
82	0.57	12	0.67	1	0.39	56	0.58	5	0.46	39	0.74
41	0.56	32	0.64	9	0.39	90	0.57	17	0.45	43	0.73
85	−0.40	57	−0.56	42	−0.40	66	−0.25	50	−0.13	80	−0.40
50	−0.46	4	−0.57	39	−0.40	83	−0.27	46	−0.18	47	−0.47
46	−0.48	85	−0.61	43	−0.42	53	−0.28	4	−0.18	46	−0.53
57	−0.48	50	−0.66	22	−0.45	4	−0.28	57	−0.19	57	−0.58
4	−0.49	46	−0.69	37	−0.55	69	−0.39	14	−0.19	4	−0.60
**73) OX1**		**74) OX2**		**75) OT**		**76) SST1**		**77) SST2**		**78) SST3**	
83	0.69	53	0.54	68	0.81	42	0.72	99	0.77	100	0.78
53	0.67	41	0.53	41	0.80	62	0.66	49	0.71	82	0.73
75	0.64	8	0.52	53	0.75	39	0.64	85	0.70	24	0.69
19	0.63	73	0.50	82	0.72	30	0.62	34	0.59	79	0.66
41	0.57	38	0.48	32	0.70	65	0.61	80	0.57	40	0.66
1	−0.39	92	−0.35	98	−0.59	1	−0.41	12	−0.50	34	−0.47
85	−0.45	50	−0.36	49	−0.67	46	−0.43	93	−0.50	77	−0.49
46	−0.48	77	−0.39	50	−0.68	50	−0.46	66	−0.51	46	−0.49
50	−0.51	98	−0.41	46	−0.69	57	−0.65	95	−0.53	49	−0.53
98	−0.53	85	−0.43	85	−0.73	4	−0.68	100	−0.54	85	−0.59
**79) SST4**		**80) SST5**		**81) NK1**		**82) NK2**		**83) NK3**		**84) TRHR**	
24	0.67	50	0.61	40	0.66	100	0.77	73	0.69	16	0.52
78	0.66	85	0.59	27	0.59	78	0.73	53	0.64	67	0.51
100	0.60	46	0.58	94	0.57	75	0.72	75	0.64	32	0.51
82	0.58	77	0.57	12	0.57	40	0.65	82	0.64	75	0.51
2	0.54	4	0.56	82	0.57	83	0.64	41	0.59	83	0.50
77	−0.17	18	−0.43	4	−0.36	4	−0.42	49	−0.54	49	−0.38
57	−0.18	23	−0.44	57	−0.36	50	−0.44	46	−0.54	50	−0.38
4	−0.20	38	−0.46	1	−0.40	49	−0.54	50	−0.55	85	−0.38
49	−0.25	75	−0.46	50	−0.46	46	−0.59	85	−0.57	98	−0.40
85	−0.29	36	−0.47	46	−0.49	85	−0.63	98	−0.58	46	−0.43
**85) VPAC1**		**86) VPAC2**		**87) V1a**		**88) V1b**		**89) V2**		**90) A1**	
49	0.93	29	0.80	76	0.46	6	0.63	100	0.65	3	0.72
77	0.70	56	0.74	19	0.44	61	0.55	78	0.63	98	0.59
46	0.70	30	0.67	7	0.40	31	0.48	24	0.57	70	0.57
98	0.68	55	0.62	32	0.40	58	0.43	64	0.57	43	0.53
50	0.65	22	0.62	52	0.39	96	0.38	54	0.55	39	0.53
12	−0.67	66	−0.35	50	−0.32	30	−0.22	98	−0.34	84	−0.34
41	−0.69	4	−0.36	46	−0.33	39	−0.23	77	−0.37	53	−0.34
66	−0.70	69	−0.38	57	−0.36	62	−0.25	56	−0.39	73	−0.34
75	−0.73	64	−0.40	4	−0.36	22	−0.26	85	−0.47	32	−0.36
100	−0.74	54	−0.42	47	−0.39	23	−0.26	49	−0.50	83	−0.39
**91) A2A**		**92) A2B**		**93) A3**		**94) P2Y1**		**95) P2Y2**		**96) P2Y4**	
13	0.68	9	0.57	23	0.68	68	0.77	100	0.58	64	0.57
21	0.65	99	0.51	100	0.59	32	0.63	23	0.55	24	0.52
11	0.55	22	0.46	41	0.57	27	0.63	40	0.54	79	0.47
27	0.53	56	0.45	97	0.56	21	0.58	12	0.49	89	0.44
12	0.53	49	0.44	78	0.55	81	0.57	38	0.49	54	0.43
10	−0.25	53	−0.28	99	−0.37	57	−0.45	99	−0.46	86	−0.17
55	−0.25	100	−0.31	34	−0.43	4	−0.46	34	−0.47	62	−0.18
87	−0.25	66	−0.34	49	−0.48	1	−0.48	77	−0.53	42	−0.18
19	−0.27	54	−0.34	77	−0.50	46	−0.55	49	−0.54	30	−0.19
76	−0.29	74	−0.35	85	−0.53	50	−0.61	85	−0.54	87	−0.23
**97) P2Y6**		**98) P2Y11**		**99) CB1**		**100) CB2**					
2	0.60	50	0.71	77	0.77	78	0.78				
23	0.59	85	0.68	49	0.54	82	0.77				
93	0.56	49	0.67	85	0.53	40	0.70				
15	0.49	46	0.64	92	0.51	12	0.66				
3	0.48	1	0.63	80	0.46	66	0.66				
1	−0.31	73	−0.53	93	−0.37	46	−0.49				
80	−0.36	83	−0.58	23	−0.38	34	−0.54				
44	−0.38	75	−0.59	100	−0.38	77	−0.54				
57	−0.45	53	−0.59	38	−0.45	49	−0.69				
4	−0.50	32	−0.64	95	−0.46	85	−0.74				

The receptor numbers (Table 1) are given in the left column and the correlations in the right column (e.g., mGluR1 has the strongest positive correlation with CRF1 (0.80)). Correlations with an absolute value of less than 0.34 are not significant after the Bonferroni correction (at *α* = 0.05) and should be interpreted with caution. The actual correlations may be somewhat stronger due the imperfect reliability of the expression data (the statistical “attenuation” phenomenon).

For compactness, the following abbreviations were used: GABA1 = GABABR1, GABA2 = GABABR2.

**Figure 4:**
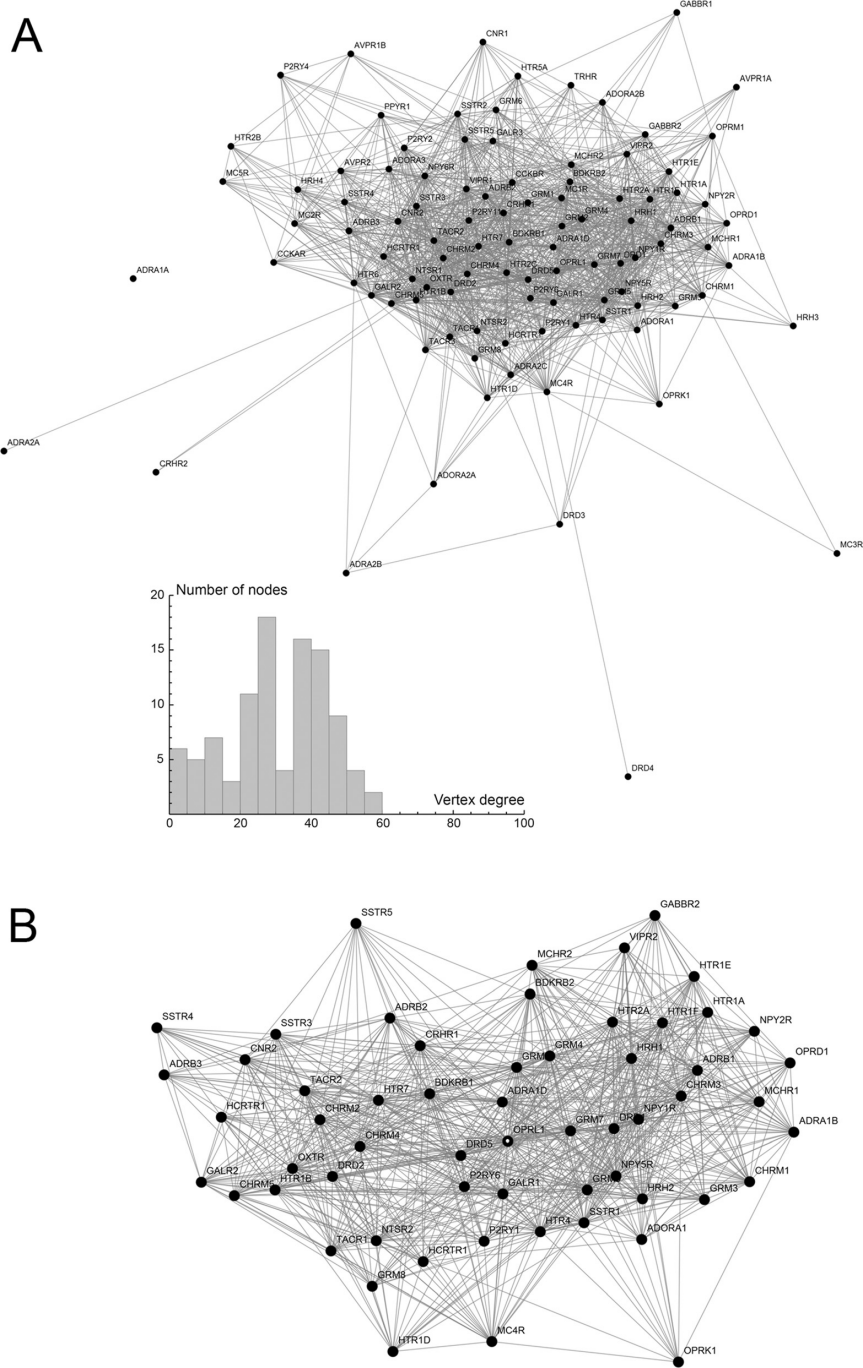
**Graph representations of receptor correlations. A**, A graph representation of all significant correlations between receptors (after the Bonferroni correction; all Cook’s distances do not exceed 1.0). The inset shows the distribution of the vertex degrees. **B**, The neighborhood subgraph of the nociceptin receptor (OPRL1); marked in the center.

All statistically significant correlations among the receptors were plotted as a graph (Figure 4A). The nociceptin receptor (ORL-1) had the highest vertex degree (connectivity), due to its significant correlations with 58 receptors (Figure 4B). The largest clique consisted of 18 completely interconnected vertices: the glutamate receptors mGluR_2_, mGluR_4_, mGluR_7_, the adrenergic receptors α_1B_, α_1D_, β_1_, the serotonin receptors 5-HT_1A_, 5-HT_1F_, 5-HT_2A_, the cholinergic receptors M_1_, M_3_, the histamine receptors H_1_, H_2_, the melanin-concentrating hormone MCH_1_, the neuropeptide Y receptors Y_1_, Y_5_, the nociceptin receptor (ORL-1), and the somatostatin receptor SST_1_. The distribution of the vertex degrees (the number of links originating in each receptor) appeared to be bimodal and did not follow the power law that is often observed in natural networks with high functional connectivity (Figure 4A, inset). However, a recent study has shown that a bimodal degree distribution can emerge in robust networks [41].

**Figure 5:**
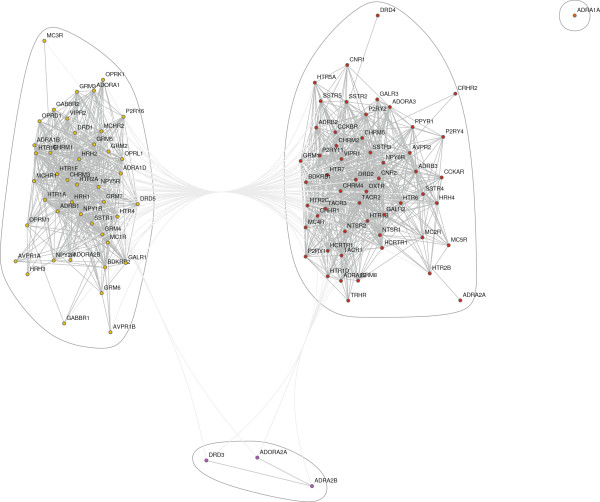
The two communities of G protein-coupled neurotransmitter receptors detected with the modularity method.

**Figure 6:**
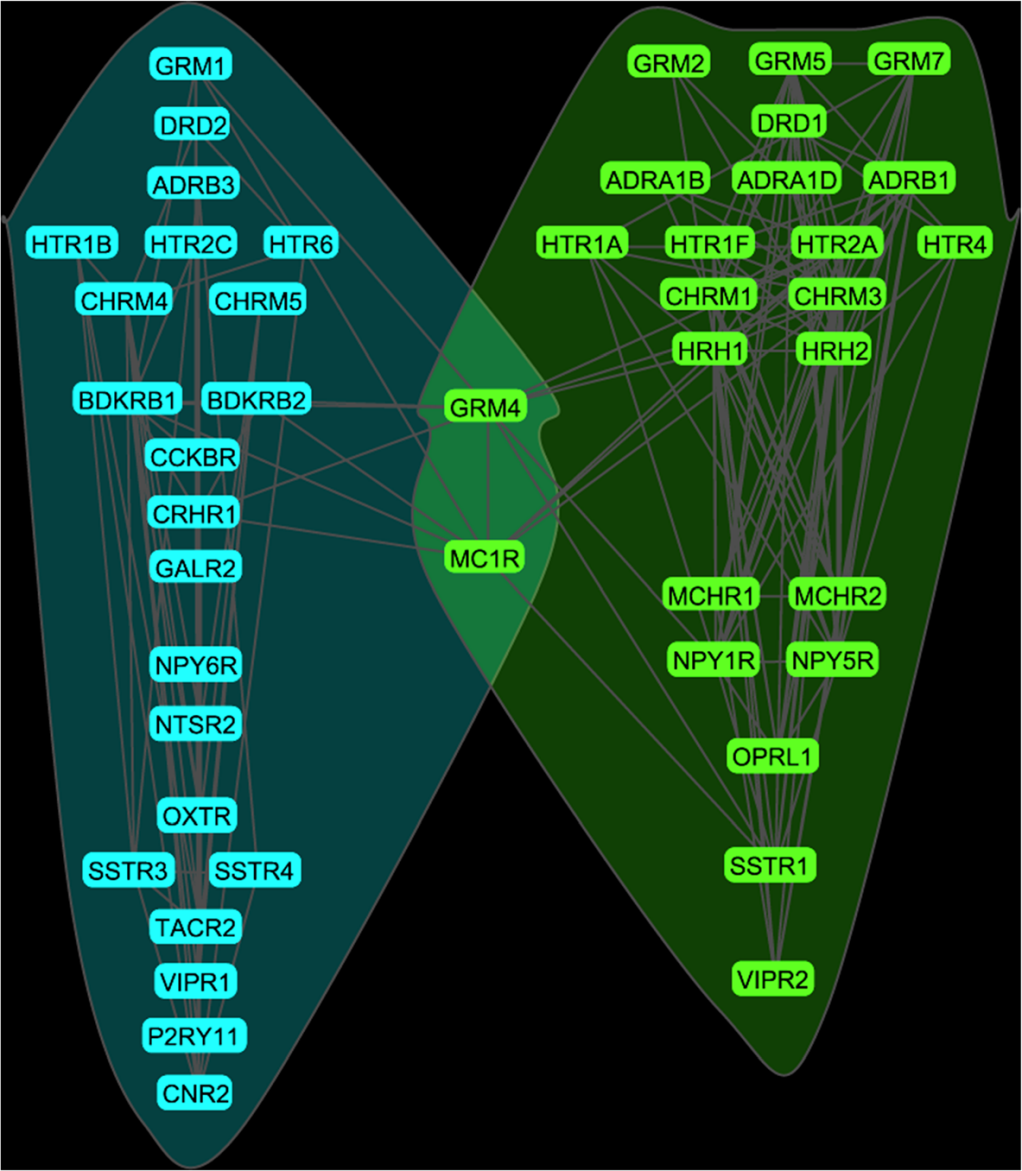
**The two communities of G protein-coupled neurotransmitter receptors detected with the clique percolation method.** The absolute values of correlations were thresholded at 0.6 (with the corresponding *p* < 10^-15^) and the clique number was set at *k* = 4.

Next, the obtained correlation information was used to examine whether some receptors groups are more tightly interlinked than other receptors or, more presicely, whether the graph (Figure 4A) can be broken down into distinct receptor communities. Two community detection methods were used: the modularity algorithm and the clique percolation algorithm [42]. The modularity method revealed two major receptor communities (Figure 5). As recommended by Palla et al. [42], the clique percolation method was optimized using several correlation thresholds (*T* = 0.5, 0.6, 0.7, 0.8, and 0.9) and *k*-cliques of several sizes. The best separation was achieved with *T* = 0.6 and *k* = 4, which again revealed two distinct receptor communities (Figure 6). With the exception of one receptor (BDKRB2), the separation among the receptors was identical to the one obtained with the modularity method. Since the clique percolation method used more stringent criteria, it excluded some more weakly correlated receptors (importantly, they were not placed in the opposite community). These analyses suggest that the human brain has two functional receptor communities, within each of which the mRNA levels are strongly correlated and can potentially affect each other. The first community contains the glutamate receptor mGluR_1_, the dopamine receptor D_2_, the adrenergic receptor β_3_, the serotonin receptors 5-HT_1B_, 5-HT_2C_, 5-HT_6_, the cholinergic receptors M_4_ and M_5_, the bradykinin receptors B_1_ and B_2_, the cholecystokinin receptor CCK_2_, the CRH receptor CRF_1_, the galanin receptor Gal_2_, the NPY receptor Y_6_, the neurotensin receptor NT_2_, the oxytocin receptor, the somatostatin receptors SST_3_ and SST_4_, the tachykinin receptor NK_2_, the VIP receptor VPAC_1_, the purine receptor P2Y_11_, and the cannabinoid receptor CB_2_. The second community contains the glutamate receptors mGluR_2_, mGluR_5_, mGluR_7_, the dopamine receptor D_1_, the adrenergic receptors α_1B_, α_1D_, β_1_, the serotonin receptors 5-HT_1A_, 5-HT_1F_, 5-HT_2A_, 5-HT_4_, the cholinergic receptors M_1_ and M_3_, the histamine receptors H_1_ and H_2_, the MCH receptors MCH_1_ and MCH_2_, the NPY receptors Y_1_ and Y_5_, the nociceptin receptor (ORL-1), the somatostatin receptor SST_1_, and the VIP receptor VPAC_2_.

## Discussion

The Allen Human Brain Atlas is a relatively new database [39,40] that continues to be updated and refined. In the absence of generally accepted standards for how gene expression data should be normalized and presented, the published expression values should be treated with caution. The expression of many receptors is highly consistent across individuals, but some receptors show a high degree of variability (Table 1). Among them is the dopamine receptor 4 (coded by the DRD4 gene), which has been extensively studied because of a functionally important polymorphism in its exon 3 [43-47]. The origin of its inconsistent distribution across individuals is not clear and may be due to either unreliable detection or true expression differences in the population. Notably, no relationship has been established between the DRD4 alleles and their mRNA levels [48].

Caution should also be exercised in the interpretation of mRNA levels that show a consistent pattern across the subjects. For many receptors, the relationship between the mRNA and protein quantities is often poorly understood, and a change in one of these measures may not indicate a change in the other. A recent large-scale study has shown that, on average, mRNA levels explain around 40% of the variability in protein levels, and that the abundance of a protein is predominantly controlled by translation [49]. In addition, many neurotransmitter receptors operate in two different signaling modes: at the membrane surface through G-proteins and, when internalized, in an arrestin-dependent fashion [50]. Neurotransmitter receptor genes can produce several mRNA splice variants, some of which may be constitutively active (ligand-independent) [51], or have different internalization properties [52]. Also, protein molecules can be phosphorylated, glycosylated, and undergo other modifications [50,53-55]. These post-translational processes place severe limitations on functional inferences from mRNA data. On the other hand, mRNA quantification allows a high degree of specificity, which remains difficult to achieve in protein analyses. Post-translational modifications of protein molecules and the absence of specific antibodies for a number of neurotransmitter receptors (contrary to the claims of manufacturers) currently do not allow large scale quantifications of proteins in the entire brain. Even though the protein data remain limited, Table 1 provides information about the possible inter-individual variability of the mRNA levels of nearly all neurotransmitter receptors and will facilitate the interpretation of completed and future studies.

The analyzed mRNA levels in brain structures reflect the cumulative gene expression in several types of neuronal and glial cells, with a possible contribution from endothelial and ependymal cells. This lack of spatial precision makes the obtained results too “coarse” for the modeling of local neural circuits. However, an association between the abundance of two receptors over many brain regions is functionally meaningful, just as biologically meaningful information can be obtained from the correlation between the population numbers of two species across geographic areas (even if the species do not directly interact). Hypofunction or hyperfunction of a receptor in a class of cells is likely to affect the activity of their local neuroecosystems, which may induce changes in the expression of receptors in other cells [56]. Therefore, estimates of the most likely associations among receptors are important for the interpretation of receptor knockout models, as well as for the prediction of changes in other receptors associated with pharmacological targeting of a specific neurotransmitter receptor [57,58]. At present, no comprehensive quantitative analysis exists to facilitate these theoretical considerations, and published data are likely to be biased by hypothesis-driven approaches, funding agency priorities, and attracting nodes in researcher networks. While the current analysis is a step forward, major theoretical breakthroughs can be expected when technical capabilities become sufficiently advanced to dynamically monitor entire receptor sets in single neurons and glial cells [35].

Currently little information is available about the correlation between the mRNA levels of receptors that form heteromeric complexes. Among them, the complex between the metabotropic glutamate receptor mGluR_2_ and the serotonin receptor 5-HT_2A_ has been particularly well studied, partly because of its potential importance in schizophrenia and other related brain disorders [32,59,60]. It has been recently reported that the disruption of 5-HT_2A_ receptor-dependent signaling can suppress mGluR_2_ transcription through epigenetic modifications in the mGluR_2_ gene promoter [61]. The present analysis found a highly significant positive correlation between the mRNA levels of these two receptors (0.49). However, mGluR_2_ had the highest positive correlations with the nociceptin receptor (ORL-1), the adrenergic α_1D_ and β_2_ receptors, the histamine H_2_ receptor, and the purine P2Y_6_ receptor; and the 5-HT_2A_ receptor had the highest positive correlations with the histamine H_1_ receptor (Figure 1), the serotonin 5-HT_1F_ receptor, the muscarinic cholinergic M_3_ receptor, and the melanin-concentrating hormone receptors MCH_1_ and MCH_2_ (Table 2).

Two receptor communities were extracted from the data (Figures 5 and 6). There are many neural circuits where these receptors interact, but it remains unclear whether the entire communities can be assigned a biologically meaningful role. It should be noted that many of the receptors in the two “minimal” communities (Figure 6) control global brain functions, such as wakefulness and sleep [62]. The two communities can be differentially affected in some brain disorders. For example, the 5-HT_1A_, 5-HT_2A_ and 5-HT_4_ receptors belong to the same community (Figure 6) and are expressed by neurons in the medial prefrontal cortex (mPFC) that project to the dorsal raphe nucleus and control serotonin release [63,64]. Altered activity of these neurons has been implicated in mood disorders [63,65,66]. The exact structure of the communities is likely to become more refined as more data become available in the Allen Brain Atlas. In general, receptor network analyses hold great promise for understanding the brain in health and disease, as has been demonstrated by recent research [36,67-69].

The relatively simple distribution of correlations (Figure 3) raises interesting questions. Theoretically, such a distribution can be obtained from a single dynamical interaction. Depending on the numerical values of its coefficients, the same process can produce uncorrelated or highly correlated equilibrium values, even if the two receptors are strongly dynamically coupled [70]. Since theoretically all receptors can be expressed by all brain cells and they can only differ in their equilibrium levels (some mRNA amounts may be undetectably small), such qualitative uniformity remains an intriguing possibility.

## Conclusions

Progress in neuroscience requires both accurate factual observations and complexity reduction. Since information processing in the brain depends on thousands of unique neurotransmitter-receptor interactions, understanding how these neurotransmitter-receptor pairs operate in functional groups is not only a theoretical imperative, but also a practical necessity. The obtained results suggest that the apparent complexity of neurotransmitter signaling has an underlying global structure, which is not readily detectable if receptor interactions are studied in isolation.

## Methods

The human brain expression data of one hundred G protein-coupled neurotransmitter receptors (Table 1) were downloaded from the Allen Brain Atlas data portal (http://human.brain-map.org; the data release of March 7, 2013). Technical details about the brain donors, tissue preparation, specificity controls, and data normalization (including normalization across brains) are described in the Allen Human Brain Atlas Technical White Papers (Case Qualification and Donor Profiles, Microarray Survey, Microarray Data Normalization).

The normalized mRNA amounts in 169 brain regions were obtained from six available subjects (three Caucasian males (31, 55, and 57 years of age), two African-American males (24 and 39 years of age), and one Hispanic female (49 years of age)).

Of the analyzed regions, the 14 regions from the myelencephalon were the central glial substance, the arcuate nucleus of the medulla, the inferior olivary complex, the gracile nucleus, the cuneate nucleus, the raphe nuclei of the medulla, the central medullary reticular group, the lateral medullary reticular group, the gigantocellular group, the hypoglossal nucleus, the dorsal motor nucleus of the vagus, the spinal trigeminal nucleus, the vestibular nuclei, and the cochlear nuclei. The 12 regions from the pontine tegmentum were the pontine nuclei, the superior olivary complex, the central gray of the pons, the paramedian pontine reticular formation, the locus ceruleus, the nucleus subceruleus, the pontine raphe nucleus, the medial parabrachial nucleus, the lateral parabrachial nucleus, the facial motor nucleus, the abducens nucleus, and the trigeminal nuclei. The 27 regions from the cerebellum were 12 vermal areas (I-II, III, IV, V, VI, VIIAf, VIIAt, VIIB, VIIIA, VIIIB, IX, X), 11 lobules (III, IV, V, VI, Crus I, Crus II, VIIB, VIIIA, VIIIB, IX, X), and the four deep cerebellar nuclei (the fastigial nucleus, the globose nucleus, the emboliform nucleus, and the dentate nucleus). The 14 regions from the mesencephalon were the ventral tegmental area, the substantia nigra, the red nucleus, the central gray of the midbrain, the midbrain raphe nuclei, the midbrain reticular formation, the trochlear nucleus, the oculomotor nuclear complex, the Edinger-Westphal nucleus, the inferior colliculus, the superior colliculus, the pretectal region, the interstitial nucleus of Cajal, and the nucleus of Darkschewitsch. The 11 regions from the thalamic area were the subthalamus, the ventral thalamus, the posterior group of nuclei, the medial geniculate complex, the dorsal lateral geniculate nucleus, the dorsal division of the lateral group of nuclei, the ventral division of the lateral group of nuclei, the anterior group of nuclei, the medial group of nuclei, the caudal group of intralaminar nuclei, and the rostral group of intralaminar nuclei. The 19 regions from the epithalamus and the hypothalamus were the pineal, the habenular nuclei, the paraventricular thalamic nuclei, the posterior hypothalamic area, the lateral hypothalamic area, the mammillary region of the lateral hypothalamic area, the mammillary body, the supramammillary nucleus, the tuberomammillary nucleus, the tuberal region of the lateral hypothalamic area, the lateral tuberal nucleus, the perifornical nucleus, the ventromedial hypothalamic nucleus, the dorsomedial hypothalamic nucleus, the anterior hypothalamic area, the arcuate nucleus of the hypothalamus, the preoptic region, the paraventricular nucleus of the hypothalamus, and the supraoptic nucleus. The 9 regions from the striatum, pallidum, and septum were the head of the caudate nucleus, the body of the caudate nucleus, the tail of the caudate nucleus, the nucleus accumbens, the putamen, the external segment of the globus pallidus, the internal segment of the globus pallidus, the substantia innominata, and the septal nuclei. The 6 regions from the amygdala were the central nucleus, the basomedial nucleus, the cortico-medial area, the basolateral nucleus, the lateral nucleus, and the amygdaloid transition zone. Two other regions from the lateral pallium were the claustrum and the piriform cortex. The 7 regions from the hippocampal formation were the parahippocampal gyrus, the dentate gyrus, the CA1 field, the CA2 field, the CA3 field, the CA4 field, and the subiculum. The cingulate gyrus was subdivided into the frontal, parietal, and retrosplenial parts, and the insula was subdivided into the short and long gyri. The 9 regions from the temporal lobe were the temporal pole, planum polare, the transverse gyri, Heschl’s gyrus, the planum temporale, the superior temporal gyrus, the middle temporal gyrus, the inferior temporal gyrus, and the fusiform gyrus. The 6 regions from the occipital lobe were the occipital pole, the cuneus, the lingual gyrus, the superior occipital gyrus, the inferior occipital gyrus, and the occipito-temporal gyrus. The 6 regions from the parietal lobe were the precuneus, the posterior paracentral lobule, superior parietal lobule, the angular gyrus, the supramarginal gyrus, and the postcentral gyrus. The 19 regions from the frontal lobe were the paraterminal gyrus, the subcallosal gyrus, the parolfactory gyri, the anterior paracentral lobule, the precentral gyrus, the superior frontal gyrus, the middle frontal gyrus, the frontal operculum, the opercular part of the inferior frontal gyrus, the triangular part of the inferior frontal gyrus, the orbital part of the inferior frontal gyrus, the lateral orbital gyrus, the medial orbital gyrus, the posterior orbital gyrus, the anterior orbital gyrus, the gyrus rectus, the superior rostral gyrus, the inferior rostral gyrus, and the frontal pole. In addition, the expression data from the cingulum, the corpus callosum, and the choroid plexus of the lateral ventricle were used.

Expression data were available from four or more subjects in 86% of the 169 brain regions, and 53% of the 169 brain regions were represented by all six subjects. Only four brain regions (2%) were represented by a single subject. The median number of mRNA probes per gene was 3. One gene was analyzed with only one probe (ADORA2B) and one gene was analyzed with 89 probes (CNR1). Probes were located on different exons as much as possible when multiple probes were used for a gene (Allen Human Brain Atlas Technical White Paper: Microarray Survey).

The data were analyzed in Mathematica 9 (Wolfram Research, Inc.). The mean expression values of each brain region of each subject were obtained by averaging all probes, and the inter-subject variability was assessed by calculating the cross-correlations between all subject pairs (15 correlations; the median values are given in Table 1). Next, the overall mean values of each brain region were obtained by averaging all available subjects, and the correlations between all unique receptor pairs (4950) were calculated. In all correlation calculations, all 169 brain regions were used as the data points. For this sample size, a correlation of ±0.16 is significant with *p* < 0.05 and a correlation of ±0.34 is significant with *p* < 0.05/4950 (i.e., it is significant after the Bonferroni correction for the multiple tests).

In graph analyses, two receptors were considered to be connected by an edge only if their correlation was significant after the Bonferroni correction (i.e., if *p* < 0.05/4950) and, additionally, if all Cook’s distances in the linear regression model did not exceed 1.0 (to avoid the effect of influential outliers).

Functional receptor communities were analyzed using the modularity algorithm implemented in Mathematica 9 and the clique percolation method implemented in CFinder (http://www.cfinder.org) and based on a published algorithm [42].

The Mathematica notebooks are available in Additional file 1 and Additional file 2.

## Competing interests

The author declares that he has no competing interests.

## Supplementary Material

Additional file 1 **The analysis of a single receptor.** The code to process the mRNA expression data of a single neurotransmitter receptor. Click here for file

Additional file 2 **The analysis of all receptors.** The code to analyze the mRNA expression associations among all neurotransmitter receptors. Click here for file
